# Laser-Based Therapies in Rosacea: A Comprehensive Review of Mechanisms, Clinical Efficacy, and Future Directions

**DOI:** 10.3390/jcm15051771

**Published:** 2026-02-26

**Authors:** Jagoda Szwach, Maciej Szwajkowski, Julia Makówka, Jakub Pyrkosz, Magdalena Łyko, Kinga Grzech-Leśniak, Alina Jankowska-Konsur

**Affiliations:** 1 Faculty of Medicine, Wroclaw Medical University, 50-367 Wroclaw, Poland; jagoda.szwach@student.umw.edu.pl (J.S.); maciej.szwajkowski@student.umw.edu.pl (M.S.); julia.makowka@student.umw.edu.pl (J.M.); jakub.pyrkosz@student.umw.edu.pl (J.P.); 2 University Centre of General Dermatology and Oncodermatology, Wroclaw Medical University, 55-556 Wroclaw, Poland; alina.jankowska-konsur@umw.edu.pl; 3 Department of Integrated Dentistry, Faculty of Dentistry, Wroclaw Medical University, 50-425 Wroclaw, Poland; kinga.grzech-lesniak@umw.edu.pl; 4 Department of Periodontics, School of Dentistry, Virginia Commonwealth University, Richmond, VA 23284, USA

**Keywords:** rosacea, laser therapy, intense pulsed light therapy

## Abstract

Rosacea is a chronic inflammatory skin disease characterized by erythematous, papular, and pustular lesions. Treatment for rosacea is tailored to the type and severity of lesions and the individual needs of the patient. The primary therapy involves topical and systemic treatments. Laser therapy is also an effective method. This review summarizes current knowledge on the application of pulsed dye lasers (PDLs), potassium titanyl phosphate (KTP) lasers, intense pulsed light (IPL), neodymium-doped yttrium aluminum garnet (Nd:YAG) lasers, carbon dioxide (CO_2_) lasers, and erbium-doped yttrium aluminum garnet (Er:YAG) lasers in the treatment of rosacea. Research confirms that the PDL remains the gold standard, demonstrating excellent clinical efficacy. The KTP and IPL lasers provide comparable outcomes, with relatively fewer adverse effects. Due to its greater depth of penetration, the Nd:YAG laser is used to treat lesions in the deeper layers of the skin. In advanced forms of rosacea, such as rhinophyma, ablative lasers, including CO_2_ and Er:YAG, are employed. This review summarizes the mechanisms of action, therapeutic applications, and adverse effects associated with the use of various laser types in the management of rosacea.

## 1. Introduction

Laser-based technologies are now widely used across dermatology and dentistry due to their anti-inflammatory, antimicrobial, and tissue-modulatory properties. Studies have confirmed that Nd:YAG and Er:YAG irradiation can reduce pathogenic biofilms and modulate inflammatory responses [1,2]. At the same time, diode and CO_2_ lasers support wound healing and microbial control in clinical settings [3,4,5]. These biological effects further justify the growing interest in laser applications for inflammatory dermatoses such as rosacea.

Rosacea is a chronic inflammatory condition characterized by facial erythema, telangiectasia, papules, and pustules [6]. Beyond its cutaneous manifestations, rosacea is associated with a substantial psychological burden, including impaired quality of life, social anxiety, and reduced self-esteem [7].

The estimated global prevalence is 5.5% [8]. However, the reported prevalence of rosacea varies widely, ranging from 0.09% to 22% [9]. Prevalence rates depend on various factors, such as geographical location, study methodology, and differences in individuals’ self-awareness of the disease [9,10].

The pathogenesis of rosacea is complex and involves the dysregulation of innate immunity, neurovascular signaling, and the cutaneous barrier function. Approximately 30–40% of rosacea patients have an affected first-degree relative, suggesting a genetic contribution to immune and vascular reactivity. One recognized etiological factor is *Demodex folliculorum*, which activates the innate immune system through upregulation of Toll-like receptor 2 (TLR-2). This activation increases kallikrein 5 activity and promotes aberrant processing of cathelicidin into proinflammatory LL-37 peptides, leading to downstream inflammatory and vasodilatory responses that contribute to persistent erythema [11,12,13,14].

Abnormal neurovascular regulation and enhanced activity of transient receptor potential (TRP) channels have also been implicated in rosacea, linking environmental triggers to vasodilation and inflammation [11]. Environmental triggers such as heat, capsaicin, and alcohol may also exacerbate symptoms by activating sensory nerve pathways and promoting the release of vasoactive mediators. Chronic inflammation promotes the overexpression of vascular endothelial growth factor (VEGF), leading to angiogenesis and increased vascular permeability. This persistent vascular remodeling underlies fixed erythema and telangiectasia, providing a biological rationale for vascular-targeted laser therapies [6].

In summary, environmental triggers and microbial stimuli activate TLR-2 signaling, leading to aberrant cathelicidin processing and sustained inflammatory responses. Mast cell activation, cytokine release, oxidative stress, and VEGF-mediated angiogenesis further promote vascular hyperreactivity and tissue remodeling. These interrelated mechanisms contribute to persistent erythema, telangiectasia, inflammatory lesions, and, in advanced cases, phymatous changes [11,15,16].

Considering the multifactorial pathogenesis of rosacea, laser therapy may target several key pathogenic components simultaneously. By reducing pathological vasculature, modulating inflammatory signaling, promoting dermal remodeling, and potentially influencing microbial load, laser-based technologies may not only improve clinical symptoms but also interfere with underlying disease mechanisms. This multimodal biological impact provides a rationale for their increasing role in comprehensive rosacea management.

Based on dominant symptoms and location, rosacea may be classified into subtypes, including erythematotelangiectatic, papulopustular, phymatous, and ocular [6]. It is worth noting that patients may present with multiple subtypes simultaneously or suffer from other dermatoses. Therefore, treatment should be based on the patient’s primary complaints and symptom severity [9,17,18]. A range of treatment modalities is available for managing rosacea, including topical therapy, oral pharmacology, and laser therapy. Metronidazole, azelaic acid, ivermectin, and calcineurin inhibitors are topical agents used for treatment [6,19,20]. Antibiotics such as tetracycline, macrolides, and metronidazole, as well as vitamin A analogue isotretinoin, are oral pharmaceuticals used mainly to treat papulopustular rosacea [20]. Laser-based therapies for rosacea primarily treat telangiectasia and erythema [21].

Leon Goldman was a pioneer of laser dermatology. In 1963, he used a ruby laser to treat various skin pathologies [22]. Since then, lasers have found numerous applications in dermatology, including tattoo removal, treatment of vascular lesions and hypertrophic scars, and aesthetic dermatology [23].

This review aims to summarize the current evidence on laser-based therapies for the management of rosacea. It will provide an overview of the types of lasers used (Table 1), their mechanisms of action, clinical efficacy, and safety. By consolidating current knowledge and highlighting areas for further investigation, this review seeks to inform dermatology practitioners about the use of laser technology in the management of rosacea.

## 2. Methods

This narrative review was based on a structured literature search conducted in July 2025 using the PubMed, Scopus, and Web of Science databases. The following search terms were applied: “rosacea” and (“laser” or “light” or “photodynamic therapy”). The term “laser” was used broadly to encompass a range of laser- and light-based devices, thereby increasing the search’s sensitivity.

Eligible publications included randomized and non-randomized clinical trials, prospective and retrospective studies, and case series evaluating laser- and light-based treatments such as PDL, IPL, KTP, Nd:YAG, Er:YAG, and CO_2_ lasers. Studies published in English or with an English abstract were considered, regardless of publication date. Articles addressing facial telangiectasia and rhinophyma were also included. The reference lists of all relevant articles were manually screened to identify additional eligible studies. The physical parameters and main clinical indications of selected laser and light-based devices are summarized in Table 1.

## 3. Lasers in the Treatment of Rosacea

### 3.1. PDL

The Pulsed Dye Laser (PDL) is a dye-based laser first approved by the FDA in 1986 for the treatment of vascular skin disorders [24], and it is now considered the gold standard for managing vascular skin conditions, including rosacea [25]. Firstly, the PDL emitted light at a wavelength of 577 nm. However, it was subsequently modified to produce wavelengths of 585 nm and 595 nm, enabling slightly deeper penetration into vascular structures [26]. Emitting light in the yellow spectrum, PDL wavelengths are selectively absorbed by oxyhemoglobin within blood vessels. The absorbed light energy is then converted into heat, leading to coagulation and closure of the targeted vessels without damaging the surrounding skin. The pulsed nature and specific wavelength of the PDL allow adequate epidermal penetration and targeted treatment of superficial vasculature, thereby minimizing the risk of complications [27,28].

In a study by S. M Clark et al. [29] on 12 patients, the effectiveness of PDL treatment for rosacea was evaluated. Treatments were given every 8 weeks. All patients tolerated the treatment well. Energy doses ranged from 5.5 to 7.5 J/cm^2^. When clinical improvement occurred, the same energy dose was maintained; if no improvement appeared, the dose was increased by 0.5 J/cm^2^. Eleven out of 12 patients experienced improvement in redness and telangiectasia after an average of 3 sessions. No significant adverse effects were observed; post-inflammatory hyperpigmentation occurred and resolved after about 3 months.

Another study by Bo Young Kim et al. [30], which compared the effectiveness of PDL and IPL therapy for rosacea, also demonstrated the clinical efficacy of PDL therapy. The treatment consisted of 4 sessions spaced 3 weeks apart, with pulse durations of 1.5 ms and energy of 8 mJ/cm^2^ for both IPL and PDL. Effectiveness was measured by reduction in telangiectasia, papules, pustules, and redness. Based on dermatologists’ subjective evaluations, eight patients (88.9%) treated with IPL showed over 50% clinical improvement, while all patients (100%) treated with PDL showed clinical improvement. No long-term adverse reactions occurred. Most patients (77.8%) reported only transient redness and swelling, which resolved within a few hours. No purpura was noted, likely due to the treatment parameters.

Findings from E. Bubul Baskan et al. [31] further demonstrate PDL’s clinical effectiveness in the treatment of rosacea. According to clinical evaluations, nine patients (64.3%) achieved clinical improvement of over 50%. Patients rated the improvement as good or excellent in 11 cases (78.5%). Quality of life increased significantly after treatment. Adverse effects were mild and temporary; purpura occurred in only 21.4% of patients. The main drawback of PDL therapy seems to be the risk of post-treatment purpura, linked to short pulse duration, causing rapid tissue overheating and blood vessel rupture. Purpura may last 1 to 2 weeks.

Even though in the study by S.R. Tan et al. [32], treatment of erythematotelangiectatic rosacea with 595 nm PDL (1.5 ms) was associated with purpura in all patients and, in some, with transient hyperpigmentation and crusting, patients reported a significant improvement in quality of life. Burning, itching, dryness, swelling, and sensitivity decreased by at least 57.1%. Six women mentioned a reduced need to cover lesions with makeup. S.R. Tan [33] obtained similar results in another study on 40 patients treated with PDL for rosacea. Although both purpura-inducing and non-purpura PDL therapies improve telangiectasia, Alam’s study showed that a single session of purpura-inducing therapy achieves much better results than non-purpura therapy. Non-purpura therapy may be suitable for minor corrections, while purpura induction is more effective for thick telangiectasia. Patients with severe telangiectasia treated with purpura induction had a mean density decrease from 3.5 to 1.8 (a reduction of 1.7), while non-purpura treatment resulted in a slight decrease of 0.4.

In people with darker skin, higher melanin content increases the risk of burns and pigment disorders like hyper- or hypopigmentation. For this reason, PDL use is limited in patients with Fitzpatrick skin types IV–V. A PDL provides significant and lasting clinical improvement. The benefits of this laser outweigh its drawbacks [34,35].

Based on available clinical studies, pulsed dye laser (PDL) therapy is considered a safe and highly effective treatment for rosacea, particularly for the erythematous and erythematotelangiectatic subtypes. Despite the risk of purpura or transient pigmentary disturbances, the clinical benefits of PDL therapy confirm its well-established position as one of the most effective treatment modalities for rosacea. The characteristics of PDL studies are summarized in Table 2.

### 3.2. KTP Laser

KTP is a potassium–titanium–phosphate laser with a wavelength of 532 nm, which is strongly absorbed by hemoglobin and melanin. Early KTP laser models were used to treat small telangiectasias because they had a small spot size and low energy, limiting the depth of penetration.

The development of KTP laser technology—including increased spot size, precise control of pulse parameters, and cryogenic cooling—has enabled deeper penetration of the laser beam while simultaneously protecting the epidermis from overheating. This has led to an expansion of KTP indications for the treatment of various superficial skin lesions, including rosacea [36,37,38].

Several studies have compared the clinical effectiveness and tolerability of KTP and PDLs in the treatment of facial redness and vascular lesions. Overall, both modalities demonstrate comparable efficacy in reducing telangiectasia and erythema. However, KTP laser therapy is frequently associated with higher patient satisfaction, faster recovery, and better treatment comfort [39]. Studies by Uebelhoer et al. and Nguyen et al. reported greater or similar reductions in redness with KTP than with PDL, along with reduced pain [39,40]. While Becher et al. observed more limited effectiveness of KTP in cases of isolated redness, the therapy remained well tolerated and showed a low incidence of mild, transient side effects [41]. Importantly, across studies, KTP treatment consistently lacked the prolonged purpura commonly associated with PDL, highlighting its favorable safety and recovery profile [39,40,41]. Nevertheless, variability in outcomes suggests that further research is needed to define better the role of KTP lasers in the management of facial redness.

It should be noted, however, that the KTP laser’s shorter wavelength results in greater absorption by epidermal melanin, which may limit its use in individuals with darker skin phototypes or tanned skin. For this reason, studies excluded subjects with Fitzpatrick skin types IV–VI [42].

Particular attention should be given to the new generation of KTP lasers, which combine two wavelengths—532 nm and 1064 nm—and are equipped with cryogenic cooling and support three different pulse structures. According to Eric F. Bernstein’s research [43], this device proved effective and safe for diffuse redness and telangiectasia related to rosacea. In a study of 21 patients, up to 3 treatments were administered 1 month apart. One month after treatment, a 39% overall improvement in skin condition was recorded. The laser effectively reduced both telangiectasia and redness. The lack of purpura, typical of KTP lasers, was an added advantage. Limited sample size requires further research.

Due to shorter recovery time and lower risk of side effects, KTP lasers may offer a more efficient alternative to PDL in rosacea treatment. Given the rapid development of laser technology, continued research on the effectiveness of new-generation KTP lasers in treating this chronic skin condition is essential. A comparison of studies using the KTP laser is shown in Table 3.

### 3.3. IPL Laser

IPL (intense pulsed light) is a broadband, incoherent light with wavelengths ranging from 500 to 1200 nm. This technique was officially authorized for clinical practice by the FDA in 1995 as a non-ablative method for the treatment of lower limb telangiectasias [44]. Using filters, it is possible to target specific chromophores, enabling the treatment of vascular and pigmentary skin lesions. Currently, IPL devices emitting either broadband or narrowband light (single- or dual-narrowband) are available, differing in wavelength ranges. IPL with a narrow wavelength band is particularly effective in treating vascular lesions. Short pulses, as little as 0.5 ms, are highly effective in treating rosacea because they match the thermal relaxation time (TRT) of small blood vessels and use low fluence energy. Additionally, the ability to adjust multiple treatment parameters, such as the number of pulses in a sequence and the interval between them, ensures high precision and flexibility in therapy [45,46].

The mechanism of IPL action is based on the selective photothermolisis of blood vessels using light energy. A wide variety of IPL devices with different parameter settings are available on the market, which can affect their efficacy and safety profiles. Multiple advantages make IPL a favorable choice. Thanks to its longer wavelength and extended pulse duration, IPL allows gradual, uniform heating of blood vessels without causing direct damage, enhancing treatment safety. Additionally, IPL enables faster treatment of larger skin areas, making the procedure more time-efficient. Its flexibility and ease of use make it an attractive therapeutic option [38,47,48].

Multiple studies support the effectiveness and safety of intense pulsed light (IPL) therapy in the treatment of vascular and pigmentary skin conditions, including rosacea, telangiectasia, hyperpigmentation, and poikiloderma. Retrospective and prospective analyses report clinical improvement rates, with excellent or satisfactory outcomes in the majority of treated patients and sustained results during long-term follow-up [49,50]. IPL therapy demonstrates a favorable safety profile, with side effects generally limited to mild, transient erythema, swelling, or occasional pigmentary changes that resolve spontaneously [50,51].

A comparative study by Neuhaus et al. [52] indicates that IPL achieves clinical outcomes comparable to pulsed dye laser (PDL) therapy in reducing facial redness and telangiectasia. While both modalities were similarly effective, patient-reported outcomes favored PDL due to less pain and discomfort than with IPL. Nevertheless, further studies are necessary due to the limited number of patients. Nevertheless, further studies are necessary due to the limited number of patients.

The ability to adjust wavelengths and parameters makes IPL a versatile device for treating both vascular and pigmentary lesions. Minimal side effects and prevention of disease recurrence make it an attractive method for treating rosacea. Attention is necessary regarding reports of treatment pain and the wide range of settings that require proper knowledge and experience. In the hands of unqualified personnel, improper parameter selection may lead to serious adverse effects.

It is important to note that photobiomodulation relies on strictly dose-dependent, non-thermal mechanisms of mitochondrial chromophore activation. Even slight deviations in treatment parameters may give a biological response. The risk of burns increases particularly in procedures performed by less experienced operators and when inappropriate filters are used. Moreover, IPL devices, which emit broadband rather than monochromatic laser light, exhibit less predictable absorption in mixed tissue [53]. A comparison of studies evaluating Intense Pulsed Light therapy is presented in Table 4.

### 3.4. Nd:YAG Laser

The Nd:YAG laser was first approved by the FDA in 1986 for ophthalmic indications, specifically for posterior capsulotomy [54]. It operates at 1064 nm and features two distinct modes: Q-switched (nanoseconds) and long-pulsed (milliseconds). Consequently, it enables deeper penetration into skin tissue and greater absorption by large, deep vessels with a bluish hue [55]. Compared with shorter-wavelength lasers, it exhibits significantly lower absorption by hemoglobin, melanin, and water, enabling deeper coagulation to depths of approximately 5–6 mm within the skin [56]. In dermatology, the neodymium laser is used to treat various vascular lesions, including telangiectasias, infantile hemangiomas (both nodular and flat), reticular varices, vascular spider veins, and rosacea [57].

Clinical studies assessing the efficacy of neodymium laser therapy in the management of rosacea have demonstrated that this laser can serve as an effective therapeutic option for both erythematotelangiectatic rosacea (ETR) and papulopustular rosacea (PPR). Greater clinical improvement and higher patient satisfaction are typically observed in the treatment of the ETR subtype. In cases of PPR, it is often recommended to combine laser therapy with additional treatments, such as antibiotic therapy, particularly in more severe presentations. A clinical study evaluating the effectiveness of treatment for ETR and PPR was conducted by Ekin Mese Say et al. [58] in 2015. The study included 66 patients: 39 with ETR and 27 with PPR. Significant clinical improvement was observed in both subgroups following therapy. The mean reduction in lesion severity was 79.5% in the ETR group and 63.0% in the PPR group. Adverse events were limited to hypopigmented atrophic scars, which occurred in only two patients; no additional side effects were reported.

The efficacy of neodymium laser therapy in PPR was further confirmed in a study by J.H. Lee [59]. Thirty patients with PPR were divided into two groups. Group A (*n* = 22) received laser therapy alone while Group B (*n* = 8) received a combination of laser therapy and doxycycline. Clinical improvement was noted in both groups, with response rates of 77.3% in Group A and 87.5% in Group B.

Overall, the neodymium laser is considered an effective and well-tolerated alternative treatment modality for rosacea.

Several studies have compared the efficacy of the Nd:YAG laser with that of the PDL in the treatment of rosacea. In a study by Samar A.M. Salem [60] involving 15 patients with Fitzpatrick skin phototype III, the clinical efficacy of Nd:YAG laser treatment, as assessed by physicians, was 73.3%, compared with 53.3% for PDL. Additionally, a significant reduction in skin substance P expression was observed. Substance P plays a key role in the pathogenesis of rosacea through its pro-inflammatory and neurogenic effects.

Interestingly, another study published in the same year, conducted by Murad Alam [61], also compared these two laser modalities but reported markedly different results. This study included 14 patients diagnosed with rosacea with Fitzpatrick skin phototypes I–III. The findings demonstrated that, while both laser treatments reduced facial erythema compared to baseline, the PDL treatment series was significantly more effective than the Nd:YAG treatment series (erythema reduction measured by spectrophotometry: PDL: 8.9%; Nd:YAG: 2.5%). Subjectively, participants also rated the PDL as the more effective treatment. The discrepancy between these studies may be attributed to differences in the Nd:YAG laser parameters used. In Salem’s study, the parameters included a fluence of 22 J/cm^2^, a spot size of 18 mm, and a pulse duration of 10 ms. In contrast, in Alam’s study, the parameters were a fluence of 6 J/cm^2^, a spot size of 8 mm, and a pulse duration of 0.3 s. The variation in Nd:YAG laser settings may have been a key factor contributing to the divergent outcomes.

Manuel Antonio Campos [62] investigated the potential benefits of combining these two laser technologies. In his study, multiplexed PDL/Nd:YAG treatment was equally effective in reducing rosacea-associated erythema as PDL monotherapy. However, the multiplexed approach resulted in fewer adverse effects, such as pain and purpura, and was rated more favorably by patients according to the Dermatology Life Quality Index (DLQI). Notably, 96.3% of patients reported that they would recommend this treatment modality.

Due to differences in tissue penetration and hemoglobin’s selective energy absorption, the side-effect profiles of these lasers also vary. PDL, with its shallower penetration, primarily affects the superficial skin layers and is most commonly associated with transient erythema, purpura, edema, or hyperpigmentation. In contrast, the Nd:YAG laser may lead to more serious adverse effects, including blister formation and linear skin grooves [63]. Therefore, Nd:YAG laser treatment requires careful parameter selection and cautious application. Nevertheless, it remains a valuable alternative to standard rosacea treatment protocols.

A direct comparison of the efficacy of PDL and Nd:YAG shows that the choice of technology should be closely dependent on the type of vascular lesions and the patient’s skin phototype.

The optimal approach appears to be a combination of both technologies, which, through the synergy of the two wavelengths, maximizes the therapeutic effect while minimizing adverse events.

Nd:YAG should not be first-line therapy for diffuse erythema due to lower hemoglobin absorption, but it is ideal for deeper, larger-caliber vessels. In clinical practice, PDL remains the first-line treatment for superficial erythema, whereas Nd:YAG is primarily effective for deeper and larger-caliber blood vessels or as a complementary therapy to PDL. The characteristics and clinical outcomes of Nd:YAG laser studies are summarized in Table 5.

### 3.5. CO_2_ Laser

The CO_2_ laser was first approved by the FDA for surgical indications in 1984 [64]. The carbon dioxide (CO_2_) laser emits light at a wavelength of 10,600 nm, which is strongly absorbed by water, the primary component of human skin. Owing to the skin’s high water content, the CO_2_ laser is particularly well-suited for precise, safe ablation of cutaneous tissue while also providing adequate hemostasis.CO_2_ laser technology offers smaller spot sizes, greater flexibility in tip selection, and enhanced application precision [65].

It is classified as an ablative laser, meaning that its mechanism of action involves tissue ablation, stimulation of neocollagenesis, and subsequent skin regeneration. Additionally, the CO_2_ laser induces coagulation of blood vessels.

This mechanism is particularly used in the treatment of rhinophyma, the most severe hypertrophic form of rosacea. The therapeutic effects of CO_2_ laser treatment include volume reduction, tissue sculpting, and hemostasis, all of which are essential in the management of this condition [66].

CO_2_ laser therapy is associated with long-lasting outcomes and high levels of patient satisfaction. In the qualification process for treatment, it is crucial to assess the skin phenotype according to the Fitzpatrick scale, as higher phototypes (IV–V) are associated with an increased risk of adverse effects, particularly hypopigmentation, and to provide an individualized approach to the patient [67,68,69].

The efficacy of CO_2_ laser treatment has been confirmed in a study by Madan et al. [70] involving 124 patients with rhinophyma. In the present study, a CO_2_ laser was employed in two operational modes: continuous mode (Sharplan 40C) and resurfacing mode (Silk Touch 20–40), selected according to the severity of rhinophyma. The continuous mode was primarily used for ablation of hypertrophic tissue, whereas the resurfacing mode was used for precise contouring and smoothing of the nasal surface. After 3 months, 118 patients rated the treatment outcome as good or excellent, while only 6 reported poor results. In a long-term follow-up survey completed by 52 patients, 100% confirmed the persistence of the therapeutic effects over time. Comparable findings were reported in a retrospective analysis by Noyman et al. [71], in which 81% of patients achieved good or very good aesthetic outcomes.

A study by P.-M. Dugourd [72] compared three techniques for the treatment of rhinophyma: electrosurgery with a monopolar diathermy knife (MDK), carbon dioxide (CO_2_) laser therapy, and cold-blade excision followed by application of 30% trichloroacetic acid (TCA). The results demonstrated that optimal treatment outcomes are achieved through an individualized approach that combines different therapeutic modalities tailored to the stage of the disease. The CO_2_ laser was found to be most effective for managing mild and moderate rhinophyma. In severe cases, although the risk of complications was higher, patients still reported complete satisfaction with the results. For advanced rhinophyma, a combination of mechanical debulking techniques and CO_2_ laser therapy is recommended.

An analysis comparing long-term outcomes of CO_2_ laser treatment with the cold-blade excision technique confirmed the efficacy and safety of both methods [73]. The study assessed aesthetic outcomes using the Rhinophyma Severity Index (RHISI) and evaluated overall patient satisfaction with the treatment. CO_2_ laser therapy was shown to be an effective and versatile modality for the management of rhinophyma.

Studies indicate that ablative CO_2_ laser treatment of rhinophyma can be effectively complemented by pulsed dye laser (PDL) therapy, which efficiently reduces erythema and contributes to improved cosmetic outcomes [74].

Due to the production of significant thermal energy, CO_2_ laser therapy carries a risk of adverse effects, including scarring, hypopigmentation, erythema, and the formation of enlarged pores at the treatment site. Nevertheless, clinical studies rarely report such complications, thereby supporting the classification of CO_2_ laser therapy as a safe and established treatment modality for rhinophyma [66,67,71,75,76]. A comparison of studies evaluating the CO_2_ laser is presented in Table 6.

### 3.6. Er:YAG Laser

The Er:YAG laser was first officially approved by the FDA in 1996 for ablative skin resurfacing applications. It emits near-infrared light at a wavelength of 2940 nm. This specific wavelength is characterized by strong water absorption in tissue. The laser can operate in Short-Pulse mode (250-350 μs) as well as in Long-Pulse mode (500 μs-10 ms), the latter having received FDA clearance slightly later in 1999. The laser energy is absorbed by tissue water, inducing thermal effects and vaporization, thereby stimulating collagen production [77].

This laser is not commonly used for rosacea treatment, and only a limited number of studies have described its use for this condition.

The Er:YAG laser’s action is gentler and more precise than that of the CO_2_ laser, which explains its role as a safer, more tissue-sparing modality in the surgical correction of rhinophyma. In the first reported series, Orenstein et al. [78] treated six patients with moderate-to-severe rhinophyma using an Er:YAG laser (1.2 J/pulse, 10 Hz, up to 10 passes). All patients achieved good-to-excellent cosmetic outcomes, with complete re-epithelialization within 7–14 days. In the study by Fincher et al. [79] on six patients treated with a dual-mode Er:YAG system (25 J/cm^2^ ablation depth, 3 mm spot, ~4 passes), results were very good to excellent with full re-epithelialization by four weeks and no adverse effects. Similarly, Mathis et al. [80] conducted a study with 11 patients treated with a dual-mode Er:YAG laser (100 µm ablation + 50 µm coagulation per pulse, 7 Hz, 4 mm spot). Ten of the eleven patients achieved excellent cosmetic outcomes, while one patient had a very good result due to mild scarring following adjunctive cold-steel debulking.

In addition to single-laser approaches, combining the advantages of both ablative systems has proven beneficial. Goon et al. [81] described six patients with moderate-to-severe rhinophyma treated with a combined Er:YAG/CO_2_ laser (Er:YAG: 1.2 J/pulse, 5 mm spot; CO_2_: 10 W). This technique allowed precise tissue vaporization with excellent hemostasis, resulting in favorable cosmetic outcomes, minimal scarring, re-epithelialization within 1–2 weeks, and no recurrences during 12 months of follow-up.

These findings highlight that the Er:YAG laser, alone or in combination with CO_2_, offers highly satisfactory cosmetic and functional outcomes, shorter healing times, and a lower complication rate compared to traditional CO_2_ monotherapy. The characteristics and clinical outcomes of Er:YAG laser studies are summarized in Table 7.

## 4. Practical Treatment Considerations 

Based on the available evidence, the following approach to laser selection for rosacea can be proposed. For diffuse erythema and superficial telangiectasias, PDL remains the method of first choice. IPL may be a suitable alternative, particularly in patients requiring treatment of large areas of the face or those with combined vascular and pigmentary problems. In cases of thicker, well-defined telangiectasia, PDL protocols that induce petechiae may provide better vessel removal. A Nd:YAG laser should be considered for deeper vessels with larger diameters, but it is generally not recommended as a first-line therapy for diffuse erythema. In rhinophyma, the treatment of choice is ablative lasers, such as CO_2_ or Er:YAG, which allow for precise tissue remodeling and volume reduction. In papulopustular subtypes, combination therapy with pharmacological agents may be indicated. The choice of treatment should be individualized based on the rosacea subtype, skin phototype, vascular characteristics, patient expectations, and tolerance of the recovery period.

## 5. Combination Therapy

Recent studies about rosacea management have underlined the effectiveness of multimodal treatments that integrate laser therapy with pharmacologic and topical agents. Combining different modalities is especially effective in severe rosacea and multiple clinical manifestations of rosacea [17,63,82]. Retrospective studies show that most patients (86%) were treated only with topical agents, of whom ¼ received a combination of topical therapy [83]. However, these medications have a similar mechanism of action, which may reduce their effectiveness. The advantage of using laser therapies in combination with other modalities lies in their ability to address different mechanisms of the pathophysiological process. For instance, a study that combined a PDL, fractional microneedling, radiofrequency, and oral 10 mg isotretinoin showed symptom improvement in recalcitrant papulopustular rosacea [63]. Therapies employing multiple modalities provide effective treatment for rosacea, and future research should focus on identifying the most effective treatment protocols. Future studies should aim to establish standardized scoring systems for erythema, telangiectasia, and patient-reported symptom improvement.

## 6. Ongoing Clinical Trials

Current clinical research aims to assess novel laser and combination therapies in patients with rosacea. An interesting clinical trial is currently being conducted at the University of Miami. Its objective is to compare the effectiveness of combined KTP laser and radiofrequency microneedling therapy with KTP laser monotherapy. The study includes patients with erythematotelangiectatic and papulopustular subtypes of rosacea. The trial is still in progress [84].

## 7. Conclusions

**Figure 1:**
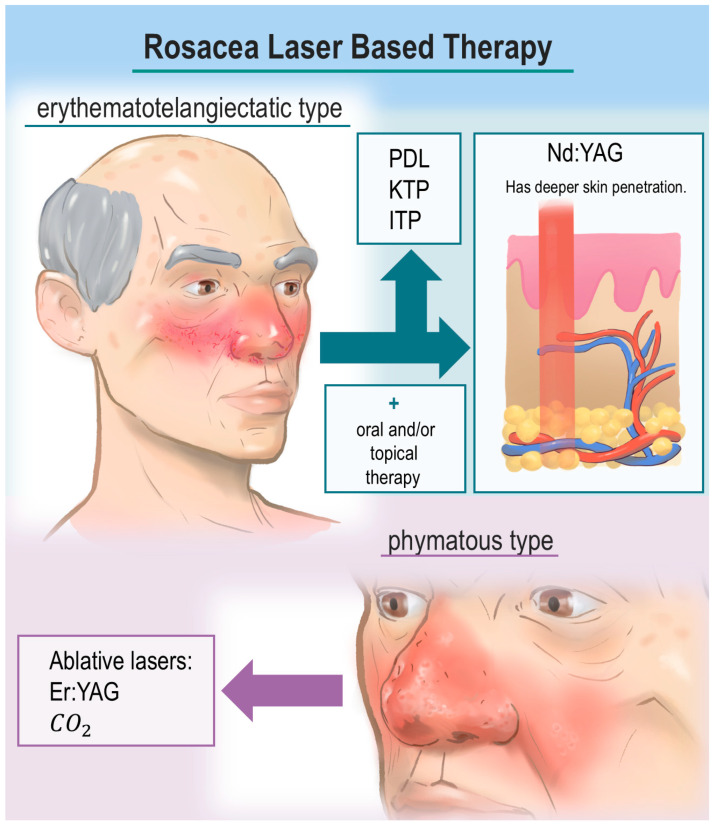
Summary of Rosacea Laser-Based Therapy.

Laser and laser-based interventions are highly effective for rosacea, offering particular benefit to patients with telangiectasia and persistent erythema. Moreover, CO_2_ and Er:YAG lasers have shown satisfactory results in the treatment of rhinophyma. PDL, KTP laser, and IPL devices have demonstrated effective and relatively safe removal of vascular lesions, while improving overall skin appearance and patients’ quality of life (Figure 1).

Lasers, thanks to their versatility, are not only used in the treatment of erythema and telangiectasia. They are also applied in cosmetic procedures such as tattoo removal and laser hair removal. Due to their effectiveness and low risk of side effects, they are widely used in cosmetology and aesthetic medicine [85,86,87].

Continuous advances in laser technology and an improved understanding of light–tissue interactions allow for increasingly precise, individualized, minimally invasive, and patient-friendly treatments. Further efforts are needed to standardize treatment protocols and outcome assessments. Future directions may include developing improved laser settings and researching new laser-based combined therapies. In summary, laser-based therapies hold significant potential to become a cornerstone of modern, comprehensive rosacea management.

## Figures and Tables

**Table 1:** Physical parameters and commonly reported clinical indications of selected laser and light-based devices.

Laser Type	Wavelength(s)	Pulse Duration	Primary Chromophore	Penetration Depth	Common Clinical Indications
PDL (Pulsed Dye Laser)	585–595 nm	0.45–40 ms	Oxyhemoglobin	Superficial–medium	Port-wine stains, rosacea, telangiectasia, spider nevi, inflammatory acne, pediatric vascular lesions
KTP (Potassium Titanyl Phosphate)	532 nm (frequency-doubled Nd:YAG)	1–20 ms	Oxyhemoglobin, Melanin	Superficial	Telangiectasia, rosacea, cherry angiomas, pigmented lesions, lentigines
IPL (Intense Pulsed Light)	500–1200 nm broadband)	2–30 ms (varies)	Oxyhemoglobin, Melanin, Water	Superficial–medium (adjustable)	Vascular and pigmented lesions, rosacea, photorejuvenation, hair removal, acne
Nd:YAG (Neodymium:Yttrium-Aluminum-Garnet)	1064 nm (long-pulsed), 532 nm (Q-switched)	ns (Q-switched), ms (long-pulsed)	Hemoglobin (1064 nm) Melanin (532 nm)	Deep (especially 1064 nm)	Leg veins, vascular lesions, tattoo removal, melasma, hair removal in darker skin types
Er:YAG (Erbium:Yttrium-Aluminum-Garnet)	2940 nm	300 μs–1 ms (ablative fractional)	Water	Very superficial	Acne scars, photodamage, melasma, epidermal lesions, skin resurfacing
CO_2_ (Carbon Dioxide Laser)	10,600 nm	0.1–1 ms (ablative fractional)	Water	Superficial–medium	Scar revision, resurfacing, warts, photodamage, rhinophyma

**Table 2:** Comparison of studies using Pulsed Dye Laser.

Author (Year)	Study Population	Laser Settings and Sessions	Rosacea Subtypes	Time of Follow Up	Outcome	Adverse Events
Clark, Lanigan, Marks (2002) [29]	12 patients	PDL 585 nm, 450 µs pulse duration, fluences 5.5–7.5 J/cm^2^, 5–7 mm spot; mean 3 sessions	Not specified	After end of treatment	50% reduction in erythema, 55% in flushing, 75% in telangiectasia overall severity decreased	Bruising hyperpigmentation mild crusting, small atrophic scars
Kim et al. (2018) [30]	30 patients	PDL 595 nm, 7 mm spot, 8–9 J/cm^2^, 6 ms pulse; 3 sessions at 4-week intervals; RF on other side	erythematotelangiectatic rosacea, papulopustular rosacea	4 weeks after last treatment	Significant improvement in rosacea severity and erythema index; RF superior to PDL for PPR lesion count	Transient erythema and edema mild pain; no scarring or pigmentation
Bubul Baskan & Belli (2018) [31]	14 patients	PDL 595 nm, spot size 7–10 mm, fluence 8–12 J/cm^2^, 10–20 ms pulse; 1–4 sessions mode 2	erythematotelangiectatic rosacea papulopustular rosacea	Mean 21.64 months	Significant improvement in erythema telangiectasia, and quality of life; benefit maintained long-term	Transient erythema pain (VAS mode 1), purpura, edema crusting; no serious side effects
Tan et al. (2004) [32]	40 patients	PDL 585 nm, 0.45 ms pulse width, fluences 5.4–7.5 J/cm^2^, spot sizes 3, 5, 7 mm; average 2.4 treatments	Not specified	Mean 23.3 months	Clinical improvement in erythema, telangiectasia, papules; high patient satisfaction	Purpura lasting 7–10 days; post-inflammatory hyperpigmentation in 6 patients; one exacerbation requiring antibiotics

PDL—Pulsed Dye Laser; VAS—visual analogue scale.

**Table 3:** Comparison of studies using Potassium Titanyl Phosphate laser.

Author (Year)	Study Population	Laser Settings and Sessions	Rosacea Subtypes	Time of Follow Up	Outcome	Adverse Events
Nguyen et al. (2024) [40]	45 patients (30 KTP, 15 PDL), Fitzpatrick I–IV	KTP: 6–10 J/cm^2^, spot 8-12 mm, 10 ms, cryogen cooling; PDL: 5–8 J/cm^2^, spot 10 mm, 0.45–2 ms	Not specified	6 weeks after last treatment	Significant decrease in erythema with both lasers; high improvement	KTP: mild-moderate redness, edema, purpura 20% (mean 1.3 days); PDL: swelling, purpura 100% (mean 6.9 days) crusting 35%, no serious AE
Becher et al. (2014) [41]	647 patients, Fitzpatrick I–IV,	KTP 532 nm: spot 1–4 mm, fluence ~11 J/cm^2^, pulse ~10 ms, ~2.8 sessions	Telangiectasia, angioma, rosacea	6 weeks after last treatment	94% clinician, 91% patient marked improvement or clearance	5.8% AE: mainly swelling crusts, blisters; 1 atrophic scar (many treatments) hypopigmentation (*n* = 2), purpura (*n* = 1)
Clark et al. (2004) [42]	204 patients, Fitzpatrick I–III	KTP 532nm: spot 1–4mm, fluence 10–14 J/cm^2^, pulse 10–14 ms, ~4 sessions for telangiectasia	Telangiectasia spider angioma	up to 2 years	Spider angioma 98%, telangiectasia 90% marked improvement	Mainly transient erythema mild hyperpigmentation (*n* = 2) 1 atrophic scar, 1 purpura, 1 blister after steroids

KTP—Potassium Titanyl Phosphate laser; PDL—Pulsed Dye Laser; AE—adverse events.

**Table 4:** Comparison of studies using Intense Pulsed Light laser. ET—Erythematotelangiectatic.

Author (Year)	Study Population	Laser Settings and Sessions	Rosacea Subtypes	Time of Follow Up	Outcome	Adverse Events
Luo et al. (2020) [50]	227 patients (107 IPL, 120 control); Fitzpatrick III–IV	IPL: 540 nm wavelength, spot size 1.5 × 4 cm^2^, fluence 10–16 J/cm^2^, pulse width 12 ms, pulse interval 10–15 ms; 3 sessions at 4-week intervals	Late-stage rosacea with persistent telangiectasia after anti-mite therapy	2 years	IPL: Effective treatment (≥90% clearance) in 66.36%; total efficacy 95.33%; improvement rate and recurrence rate significantly better than control	Mild burning (*n* = 11 cases) swelling (*n* = 8), hyperpigmentation (*n* = 3), facial blisters (*n* = 2); temporary redness/blisters subsided within 1 week, hyperpigmentation within 3 months
Neuhaus et al. (2009) [52]	29 patients (20 F, 9 M), Fitzpatrick I–III	IPL: 560 nm filter, pulse train (2.4 + 6.0 ms, 15 ms delay), starting fluence 25 J/cm^2^, 3 sessions (1 per month), split-face, fluence increased per session if tolerated.	ET rosacea	1 month after 3rd treatment (visit 4)	Significant reduction in erythema, telangiectasia, and symptoms; no significant difference between PDL and IPL;	1 patient (IPL group) excessive swelling and reaction (did not attend follow-up). No other adverse events reported.
Arminda (2024) [51]	100 patients (50 vascular, 50 pigmented), adults	Narrow-band AFT-IPL, wavelength 450–600 nm, spot 3 cm^2^, pulse 13 s, 1–4 sessions (every 3 weeks), 3-month follow-up	Vascular and pigmented lesions of face & body	3 months after last session	Mean GAIS score for vascular: 8.02/10; pigmented: 8.14/10; satisfaction: 8/10. High, comparable efficacy	2 mild, transient body burns (resolved in 8–10 weeks); no pain; no other relevant adverse events

**Table 5:** Comparison of studies using the Nd:YAG laser.

Author (Year)	Study Population	Laser Settings And Sessions	Rosacea Subtypes	Time of Follow Up	Outcome	Adverse Events
Ekin Mese Say et al. (2015) [58]	66 patients (39 ETR, 27 PPR); Fitzpatrick II–III	Nd:YAG 1064 nm, spot size 2–3 mm, fluence 100–160 J/cm^2^, pulse duration 15–20 ms, average number of sessions: 3.95 (ETR 2–8; PPR 1–10), interval 3–4 weeks	ETR, PPR	4 weeks after completing treatment	ETR: 79.49% global improvement; PPR: 62.96% improvement; in both groups, most patients reported significant, or excellent improvement.	2 patients hypopigmentation atrophic scars; no other serious adverse events occurred
J.H. Lee et al. (2015) [59]	30 patients with PPR; group A: 22 patients mild moderate PPR, laser only), group B: 8 patients (severe PPR, laser + doxycycline) Fitzpatrick IV–V	Nd:YAG (GentleMax Candela), 1064 nm, rejuvenation mode, spot size 10 mm, fluence 40–50 J/cm^2^, pulse duration 50 ms, 3 sessions every 4 weeks	PPR	4 weeks after completing treatment	77.3% (17 out of 22) in group A and 87.5% (7 out of 8) in group B	Temporary redness and mild pain during the procedure; no scars, discoloration, swelling, or purpura
Samar A.M. Salem et al. (2013) [60]	15 women, Fitzpatrick III	Nd:YAG 1064 nm (Candela GentleYAG), fluence 22 J/cm^2^, spot size 18 mm, pulse 10 ms, 3 sessions every 4 weeks	ETR	4 weeks after completing treatment	one patient (6.7%) had mild improvement, 3 patients (20.0%) had moderate improvement, and eleven patients (73.3%)	Transient erythema; no purpura, discoloration or scarring
M. Alam (2013) [61]	14 patients Fitzpatrick I–III	Nd:YAG (1064 nm, Genesis module): 6 J/cm^2^; 8 mm spot; 0.3 ms pulse. 4 sessions, 3–4 week intervals	ETR	4 weeks after completing treatment	mean 2.5% (not significant) subjectively 34% improvement	Nd:YAG associated with less pain, no bruising
Campos et al. (2019) [62]	29 patients enrolled, 27 completed	Multiplexed PDL/Nd:YAG (595 + 1064 nm): PDL 7.0 J/cm^2^ (10 ms), Nd:YAG 35 J/cm^2^ (15 ms, long delay), 7 mm spot, DCD level 3/5, 1 pass minimal overlap 3 sessions at 3–4 week intervals	ETR	4 weeks after completing treatment	Multiplexed PDL/Nd:YAG: fewer side effects, higher satisfaction (96.3% would recommend)	2 patients dropped out (purpura interfering with excessive pain)
Kwon et al. (2018) [63]	20 patients Fitzpatrick III–IV	LPNY (1064 nm, AILEEN plus): 2 mm spot, 10–25 ms pulse, 150–250 J. 3 sessions at 4-week intervals	ETR, nasal telangiectasia	4 weeks after completing treatment	Nd:YAG: better for thick dilated vessels High patient satisfaction	Nd:YAG erythema (*n* = 17), purpura (*n* = 3), pain (*n* = 11), blister (*n* = 1), linear furrow (*n* = 1)

ETR—erythematotelangiectatic rosacea; PPR—papulopustular rosacea;.

**Table 6:** Comparison of studies using CO_2_ laser.

Author (Year)	Study Population	Laser Settings and Sessions	Rosacea Subtypes	Time of Follow Up	Outcome	Adverse Events
Madan et al. (2009) [70]	124 patients mostly Caucasian	Sharplan 40C CO_2_ laser; continuous mode 10–20 W (1–3 mm beam) resurfacing mode Silk Touch 20–40 W (4–7 mm spot); mostly single session (115/124), some multiple up to 4)	Rhinophyma (end-stage rosacea) classified as minor, moderate, major	3 months post-treatment questionnaire follow-up 2–12 years	Good–excellent in 118/124, poor in 6, high long-term satisfaction improved confidence and well-being	Pain from local anaesthesia scarring (*n* = 4), hypopigmentation (*n* = 4), open pores (*n* = 2), infection with scarring (*n* = 1), notching nasal ala (*n* = 2), hypertrophic scarring in 1 Asian patient
Noyman et al. (2025) [71]	16 patients	Sharplan 40C CO_2_ laser, non-fractional continuous wave, 15–20 W, 1.2 mm focal spot, 300 mm handpiece; single-session under local anesthesia	Severe rhinophyma some with erythematotelangiectatic papulopustular rosacea (2 patients)	Follow-up at 1, 2, 4, 12 weeks main evaluation at 3 months long-term questionnaire	Good or excellent aesthetic outcome in 81%. 85% would recommend this treatment	Most none mild scarring (*n* = 1), temporary swelling (*n* = 1), pain mild moderate in most, no long-term PIH, hypertrophic or keloid scars
Dugourd et al. (2021) [72]	25 patients Fitzpatrick II–III	CO_2_ laser (CO2RE, Syneron-Candela) continuous mode 9 W for debulking pulsed mode 2–3 mJ fractional mode (fusion 40%, 90/50 mJ) for mild cases and “risk areas	Rhinophyma graded by NRS: mild, moderate, severe	Photographic evaluation at 3 months patient questionnaires	Good–excellent improvement in 80% (20/25); moderate in 16% (4/25); poor in 4% (1/25). Patient satisfaction 88% (satisfied or very satisfied	Only 1 hypertrophic scar
Amiri et al. (2025) [73]	15 patients, Fitzpatrick II–III	Lumenis Ultrapulse Active FX fractional CO_2_ laser: 125 mJ/cm^2^, 150–450 Hz, spot size 2–7, density 9; single session after medical downstaging local anesthesia (lidocaine + epinephrine)	Rhinophyma (severity scored with RHISI)	The changes in RHISI score were assessed at 23 and 17 months after	71% rated cosmetic outcome as good excellent, 86% would recommend	no major scarring pigmentary changes, or severe complications reported

NRS—numeric rating scale; PIH—post-inflammatory hyperpigmentation.

**Table 7:** Comparison of studies using Er:YAG laser.

Author (Year)	Study Population	Laser Settings and Sessions	Rosacea Subtypes	Time of Follow Up	Outcome	Adverse Events
Orenstein et al. (2001) [78]	6 patients	Er:YAG (Derma TM20), 5 mm beam, 1.2 J/pulse, single session	Rhinophyma	1 month, 1–2 years	4 excellent normal nasal contour, no scarring); 2 good (acceptable contour minimal scarring); re-epithelization 7–14 days, erythema resolved in 1 month	None reported
Fincher et al. (2004) [79]	6 patients Fitzpatrick II–IV	Dual-mode Er:YAG laser (Contour Sciton Inc.); 3 mm spot, scanner handpiece; 100 µm ablation (25 J/cm^2^) + 50 coagulation, single session	Rhinophyma	3–6 months	All patients achieved very good or excellent outcomes; re-epithelialization complete by 4 weeks; high satisfaction	None reported
Mathis et al. (2019) [80]	11 patients Fitzpatrick I–II	Dual-mode Er:YAG Joule Sciton Inc.); 2940 nm; 4 mm spot; 100 µm ablation + 50 µm coagulation per pulse; 7 Hz, single session	Rhinophyma	1 month	10/11 patients had excellent results normal contour, no scarring); 1 patient very good (mild scarring after debulking) all reported satisfaction	None reported
Goon et al. (2004) [81]	6 patients	Combined Er:YAG/CO_2_ laser; Er:YAG: 1.2 J/pulse, 5 mm spot; CO_2_: 10 W, single session	Rhinophyma	1 year	Re-epithelialization within 1–2 weeks all patients achieved good contour color alar symmetry minimal scarring all satisfied with cosmetic results	None reported

## Data Availability

No new data were created or analyzed in this study.
